# Psychosocial and digital predictors of Hepatitis B vaccination uptake in healthcare workers: insights from a Nigerian tertiary hospital

**DOI:** 10.1186/s41182-025-00893-4

**Published:** 2025-12-31

**Authors:** Stephen Olaide Aremu, Akyala Ishaku Adamu, Yakubu Boyi Ngwai, Abdillahi Abdi Barkhadle

**Affiliations:** 1 Global Health and Infectious Diseases Control Institute, Nasarawa State University, Keffi, Nasarawa State Nigeria; 2 Rift Valley University, Jimma, Ethiopia

**Keywords:** Hepatitis B, Vaccine adherence, Healthcare workers, Psychosocial, Digital belief systems, Health behavior

## Abstract

**Introduction:**

Hepatitis B virus (HBV) infection remains a significant global health threat, with over 296 million individuals living with chronic HBV and more than 820,000 annual deaths due to related complications. Healthcare workers (HCWs) are at heightened risk due to occupational exposure, especially in sub-Saharan Africa, where HBV prevalence and health system limitations compound their vulnerability. Despite the availability of an effective vaccine, adherence to the three-dose HBV vaccination schedule among HCWs in Nigeria remains suboptimal. This study seeks to explore the psychosocial and digital factors influencing non-adherence to the HBV vaccination schedule among HCWs in Nigeria, with the aim of informing more targeted and effective interventions.

**Methodology:**

A cross-sectional survey was conducted among 530 healthcare workers at FMC Keffi to assess Hepatitis B vaccine uptake and its determinants. Data were collected using a structured, pre-validated questionnaire covering vaccination status, knowledge, attitudes, barriers, and digital access. Statistical analyses included descriptive statistics, Chi-square tests, binary logistic regression, mediation (PROCESS Model 4), and moderation analyses. These methods evaluated the direct and indirect effects of knowledge, attitudes, occupational exposure, and digital intervention beliefs on vaccine adherence, with gender assessed as a moderating variable.

**Results and discussion:**

Among 530 healthcare workers at FMC Keffi, only 19.4% were fully vaccinated against Hepatitis B, while 72.6% had received at least two doses. Mediation analysis showed that knowledge had no significant direct or indirect effect on vaccine uptake (*p* = 0.21), whereas attitude was a strong predictor (*B* = − 9.85, *p* < 0.001). Occupational exposure significantly influenced adherence (χ2 = 33.00, *p* < 0.001), as none of the unexposed were fully vaccinated. Gender moderated the attitude–uptake link, with females showing stronger associations. Digital access remained low, limiting the effectiveness of digital intervention strategies.

**Conclusion:**

This study concludes that HBV vaccine adherence among healthcare workers is influenced more by attitudinal factors, occupational exposure, and gender dynamics than by knowledge alone. Digital intervention strategies remain underutilized due to limited access. To improve vaccine uptake, health systems must adopt multifaceted approaches that prioritize behavioral insights, gender sensitivity, and equitable access to digital health resources.

**Supplementary Information:**

The online version contains supplementary material available at 10.1186/s41182-025-00893-4.

## Introduction

Hepatitis B virus (HBV) infection remains a major global public health challenge, with over 296 million people living with chronic HBV infection and approximately 820,000 deaths annually attributed to HBV-related complications such as cirrhosis and hepatocellular carcinoma (HCC) [1, 2]. The World Health Organization (WHO) recognizes Hepatitis B virus (HBV) as a vaccine-preventable infection; however, the global viral hepatitis elimination goals set for 2030 extend beyond vaccination alone. They encompass a comprehensive strategy that includes effective prevention, diagnosis, treatment, harm reduction for people who inject drugs, as well as safe blood transfusion and injection practices, each with defined targets for progress [3].

Healthcare workers (HCWs) are among the most vulnerable groups to HBV infection due to their routine exposure to blood, sharps, and other potentially infectious materials [4]. In sub-Saharan Africa, where HBV prevalence exceeds 8% in some healthcare facilities, the occupational risk to HCWs is further heightened by under-resourced health systems, heavy patient loads, and frequent shortages of protective equipment [5, 6]. Nigeria, the most populous country in the region, bears a significant HBV burden, with an estimated 15–20 million chronic carriers [7].

Despite the availability and proven efficacy of the three-dose HBV vaccine, adherence among Nigerian HCWs remains low, with studies reporting full vaccination rates ranging from 18 to 25% and partial vaccination coverage of about 60–70% [2, 8]. Prior studies have documented poor completion rates, with some HCWs initiating but not completing the vaccine schedule, and others entirely unaware of their HBV status or the vaccine’s availability [8–10]. This non-adherence not only compromises individual protection, but also undermines workplace safety and contributes to the broader challenge of HBV transmission in healthcare settings [9, 10].

Multiple factors have been implicated in vaccine non-adherence, ranging from knowledge gaps and negative attitudes to perceived barriers such as cost, time constraints, and lack of institutional support [11, 12]. In recent years, the use of digital health interventions, including mobile reminders, electronic health records, and social media platforms, has emerged as a promising strategy to improve vaccine awareness and adherence [13]. However, the role of digital belief systems, especially in low-resource settings, remains poorly understood.

This study examines the psychosocial determinants of Hepatitis B (HBV) vaccine adherence among healthcare workers (HCWs) in a Nigerian tertiary healthcare facility. Specifically, it will assess knowledge, attitudes, and perceived barriers to vaccine adherence, and evaluate the mediating roles of knowledge and attitudes in the decision-making process. In addition, the study will explore the moderating effects of gender and digital belief systems on adherence behavior. It will also identify demographic predictors of adherence to inform targeted public health interventions.

The study will generate empirical evidence on vaccine behavior in this high-risk occupational group. The findings are expected to inform local and global strategies to improve vaccine adherence, safeguard healthcare workers, and contribute to HBV elimination goals in line with the World Health Organization’s (WHO) agenda. This study, therefore, seeks to bridge critical gaps in the understanding of HBV vaccine adherence among HCWs in a Nigerian tertiary healthcare facility.

## Methodology

### Study design and setting

This study employed a cross-sectional analytical design to investigate the psychosocial and systemic determinants of HepB vaccination adherence among healthcare workers (HCWs) at Federal Medical Centre (FMC), Keffi, located in Nasarawa State, North Central Nigeria. The institution is a tertiary healthcare facility serving a diverse catchment population, thus providing a representative cross-section of HCWs across clinical and non-clinical cadres. The study period spanned from March to May 2025.

Nasarawa State has been reported to have a relatively high burden of HBV infection, with prevalence estimates ranging between 12–20%, which is above the national average. This underscores the importance of HepB vaccination adherence among HCWs in the region, given their increased occupational exposure. FMC Keffi has a patient load of approximately 150,000 outpatient visits annually and a staff strength of about 4,000, resulting in a significant patient-to-HCW ratio. This workload increases the risk of accidental exposure to bloodborne pathogens, further justifying the focus on vaccination adherence.

### Participants and sampling

#### Eligibility criteria

Participants included HCWs across various professional categories (physicians, pharmacists, physiotherapists, nurses, laboratory scientists/technicians, administrative staff, and auxiliary health staff) who had been employed at FMC Keffi for at least 12 months. Inclusion criteria were:

- Age ≥ 18 years
- Currently employed at FMC Keffi for at least 12 months
- Provided informed consent.

Exclusion criteria included:

- History of chronic liver disease or prior diagnosis of HBV.

#### Sampling technique

A probability sampling technique was employed to enhance representativeness and reduce selection bias. Specifically, simple random sampling was used to ensure that every healthcare worker in the study population had an equal chance of being selected. This approach was used to strengthen the external validity of the findings and to minimize potential biases that may arise when attempting to capture the entire workforce. By applying this method, the study achieved a representative sample that supports more generalizable conclusions while maintaining the rigor needed for subgroup analyses. Of the 580 HCWs approached, 530 completed responses were included in the final dataset, yielding a response rate of 91.4%.

### Data collection instruments

A structured, pre-tested, and researcher-administered questionnaire was used to collect data. The pre-test was conducted among healthcare professionals enrolled at the Global Health and Infectious Diseases Control Institute, Nasarawa State University, Keffi (GHIDI-NSUK), to assess clarity, relevance, and reliability of the items. The instrument demonstrated good internal consistency, with a Cronbach’s alpha score of 0.87, thereby supporting its validity for use in the main study. The instrument was developed based on an extensive review of relevant literature and guided by the Health Belief Model (HBM) and Theory of Planned Behavior (TPB) frameworks [11]. Although the questionnaire was developed based on an extensive review of relevant literature and guided by the Health Belief Model (HBM) and the Theory of Planned Behavior (TPB), these frameworks have inherent limitations. The HBM, for instance, has been critiqued for focusing predominantly on individual perceptions and neglecting broader social, cultural, and environmental influences on health behavior. Similarly, while the TPB incorporates attitudes, subjective norms, and perceived behavioral control, it assumes rational decision-making and may underestimate the role of emotions, habits, and contextual constraints. These limitations should be considered when interpreting the study’s findings. The questionnaire was structured into seven sections such as Demographics and professional profile**:** age, gender, marital status, religion, education, profession, department, and years of experience.

- Occupational exposure risk: questions assessing exposure to blood, body fluids, or needlestick injuries.
- HepB vaccination history: questions assessed vaccine initiation, number of doses received, and completion of the three-dose schedule. Adherence was determined by self-reported receipt of all three recommended doses within the appropriate intervals.
- Knowledge Score (KNOWSCRE): assessed via 10-item true/false questions (Cronbach’s α = 0.81).
- Attitude Score (ATTSCORE): 8-item Likert scale items (strongly disagree to strongly agree), assessing perceived benefits, concerns, and motivation to vaccinate (Cronbach’s α = 0.84).
- Perceived Barriers Score (BARSCORE): 6-item barrier inventory including cost, access, time constraints, and vaccine hesitancy (Cronbach’s α = 0.79).
- Digital Intervention Belief (DOYOUBEL): binary item assessing whether the respondent self-reported access to digital health platforms could improve vaccine adherence.

The tool was validated through expert review by public health physicians, epidemiologists, and behavioral scientists to ensure content and face validity. In addition, a pilot pre-test was conducted among 30 healthcare professionals at the Global Health and Infectious Diseases Control Institute, Nasarawa State University, Keffi (GHIDI–NSUK) which was not included in the final analysis. Minor revisions were made for clarity and cultural context. This process helped to identify and correct issues related to comprehension, cultural appropriateness, and the potential for skipped or misinterpreted questions, particularly among auxiliary health staff.

### Operational definitions

- Full adherence to HepB vaccine: receipt of all three doses of the vaccine according to WHO guidelines.
- Partial/non-adherence: initiated but did not complete, or never received the vaccine.
- Digital access: self-reported access to any digital health platform or information related to Hep B vaccination.
- High vs low attitude: dichotomized based on the median score of ATTSCORE.

### Ethical considerations

Ethical approval was obtained from the Health Research Ethics Committee of FMC Keffi (Approval No: FMC/KF/REC/026542/24). Participation was voluntary, anonymity was ensured, and written informed consent was obtained from all respondents. The study adhered to the ethical principles outlined in the Declaration of Helsinki.

### Statistical analysis

All data were entered into IBM SPSS version 26 and analyzed using a combination of SPSS and Hayes’ PROCESS Macro (v4.0) for mediation and moderation analyses. Data screening involved checks for missing values, multicollinearity, normality, and outliers. Only completed questionnaires were retained, resulting in a final sample of *n* = *531*. The overall level of missingness across variables was low (0%). As the proportion of missing data was below the conventional 5% threshold, listwise deletion was applied. Examination of distributions revealed that specific variables, e.g., “knowledge scores” and “attitude toward vaccination” were positively skewed. To address this, a log10 transformation was applied to the knowledge scores and a square-root transformation to attitude scores, which successfully reduced skewness to within acceptable limits (± 2). Descriptive statistics, including means, standard deviations, frequencies, and percentages, were computed to summarize participant characteristics and key study variables.

### Analytical framework

A parallel mediation model (PROCESS Model 4) was applied to test the indirect effects of belief in a simulated digital intervention (X) on vaccine uptake (Y) through Knowledge Score (M1) and Barrier Score (M2). Given the absence of documented evidence of established digital interventions for Hepatitis B vaccination in Nigeria, this construct was assessed hypothetically to explore its potential influence on adherence behavior. The model estimated: direct effects of knowledge on vaccine uptake, indirect effects through attitude, total effects, bootstrapping (5000 samples) was used to generate bias-corrected confidence intervals (95% CI) for indirect effects. A mediation effect was deemed significant if the CI excluded zero.

The original plan involved testing whether digital access moderated the relationship between occupational exposure and vaccine uptake (PROCESS Model 1). However, due to singularity issues (i.e., limited variability in digital access), a formal moderation analysis was infeasible. Instead, a cross-tabulation and Chi-square test was employed to examine the association between exposure status and vaccine adherence. A binary logistic regression model was employed with vaccination status as the dependent variable and demographic variables (gender, age, marital status, religion, profession, department, education level) as predictors. Odds Ratios (Exp(B)), Wald statistics, and 95% confidence intervals were reported. Model performance was assessed using:

- Omnibus test of model coefficients
- Nagelkerke R² and Cox & Snell R²
- Classification accuracy (% correctly predicted).

A parallel mediation model (PROCESS Model 4) was applied to test the indirect effects of belief in digital intervention (X) on vaccine uptake (Y) through Knowledge Score (M1) and Barrier Score (M2). All paths (X → M1, X → M2, M1 → Y, M2 → Y) and direct effects (X → Y) were analyzed. Bootstrap confidence intervals (5000 samples) were used to test for statistical significance.

Moderation analysis (PROCESS Model 1) was conducted to evaluate whether gender moderated the relationship between attitude and vaccination adherence. The interaction term (Attitude × Gender) was entered into the model, and significance was determined via Chi-square and conditional effects. Marginal effects at different levels of gender were estimated and interpreted.

### Model assumptions and robustness checks

- Linearity: relationships between continuous predictors and log odds of the outcome were checked.
- Multicollinearity: variance inflation factor (VIF) and tolerance levels were within acceptable ranges (< 5).
- Bootstrapping: non-parametric resampling was used to strengthen inference for indirect effects.
- Goodness-of-fit: logistic regression models were evaluated using –2 log likelihood, McFadden’s R², and Nagelkerke R².
- Residual diagnostics: Cook’s distance and standardized residuals were assessed to rule out influential outliers.

### Data management and security

Data were anonymized and stored on encrypted drives accessible only to the research team. Access logs and audit trails were maintained in compliance with institutional data governance protocols. All paper-based forms were digitized and shredded post-digitization.

### Software used

- IBM SPSS Statistics v26: data management and regression analyses
- PROCESS Macro v4.0: mediation and moderation modeling
- Microsoft Excel: descriptive statistics and visualizations.

## Results

**Fig. 1:**
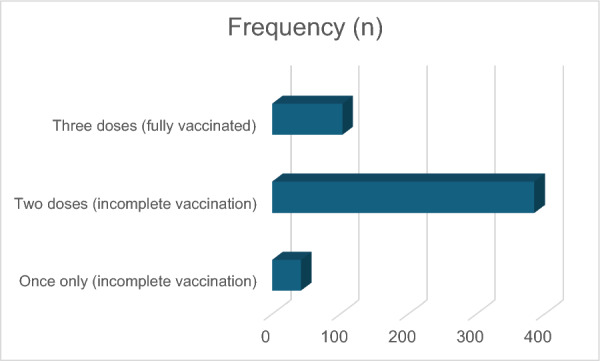
Frequency distribution of HepB vaccination doses among healthcare workers at FMC Keffi

Most healthcare workers at FMC Keffi have received at least two doses of the Hepatitis B vaccine, accounting for 72.6% of the respondents. A smaller proportion, 19.4%, reported being fully vaccinated with all three recommended doses. Meanwhile, only 7.9% indicated that they had received just a single dose, reflecting a gap in complete immunization among some individuals (Fig. 1).

The model was significant (Nagelkerke *R*^2^ = 0.8375), explaining 83.75% of the variance in vaccination uptake (Tables 1, 2, 3, 4). However, the direct effect of knowledge on vaccine uptake was not significant (*B* = 0.798, *p* = 0.2097). Furthermore, while attitude was a significant predictor of vaccination uptake (*B* = − 9.8489, *p* < 0.001), the mediation pathway from knowledge to vaccine uptake via attitude was not significant (indirect effect = − 0.4432, 95% CI [− 1.4781, 0.6816]). These results suggest that knowledge does not influence vaccination adherence through attitude in this sample. Knowledge explained virtually none of the variance in attitude (*R*^2^ = 0.0013) and had no significant effect (*p* = 0.4129).

**Table 1:** Model summary for the mediation analysis (knowledge → attitude)

Model	*R*	*R*^2^	MSE	F	df1	df2	*p*-value
Model for attitude	0.0356	0.0013	0.1508	0.6714	1	528	0.4129

**Table 2:** Model summary for the outcome (vaccine uptake)

Metric	Value
−2 Log likelihood (−2LL)	157.1032
Model log likelihood (ModelLL)	446.5397
Degrees of freedom (df)	2
p-value (overall model)	0.0000
McFadden’s *R*^2^	0.7397
Cox & Snell *R*^2^	0.5694
Nagelkerke *R*^2^	0.8375

**Table 3:** Variables in the equation for vaccine uptake

Variable	Coefficient (*B*)	Standard error (SE)	*Z*-value	*p*-value	95% Confidence interval (CI)
Constant	22.1760	3.4767	6.3784	< 0.001	[15.3618, 28.9903]
Knowledge Score (KNOWSCRE)	0.7976	0.6359	1.2544	0.2097	[− 0.4486, 2.0439]
Attitude Score (ATTSCORE)	− 9.8489	0.8477	− 11.6188	< 0.001	[− 11.5103, − 8.1875]

**Table 4:** Direct and indirect effects of knowledge on vaccine uptake (through attitude)

Effect	Coefficient (*B*)	Bootstrapped SE	95% Confidence interval (CI)
Direct effect	0.7976	0.6359	[− 0.4486, 2.0439]
Indirect effect (through attitude)	− 0.4432	0.5494	[− 1.4781, 0.6816]

The indirect pathway from knowledge → attitude → vaccine uptake is not significant.

The results showed that none (0%) of the healthcare workers who reported no occupational exposure were fully vaccinated. In contrast, 30.3% of those who had experienced occupational exposure had completed the full HBV vaccination series. This suggests a strong positive relationship between occupational exposure experience and vaccine uptake (Tables 5, 6). Exposure to risk may therefore act as a motivating factor for completing the vaccination schedule.

**Table 5:** Association between occupational exposure risk and completion of Hepatitis B vaccination among healthcare workers

Exposure risk	Vaccination status		Total (*n*, %)
	Incomplete (*n*, %)	Fully vaccinated (*n*, %)	
No exposure	81 (100.0%)	0 (0.0%)	81 (15.3%)
Exposure	313 (69.7%)	136 (30.3%)	449 (84.7%)
Total	394 (74.3%)	136 (25.7%)	530 (100.0%)

*p* < 0.001

**Table 6:** Omnibus test and model summary

Test	Value	Df	*p*
Omnibus Chi-square (model)	594.645	7	< 0.001
–2 Log likelihood	8.998		
Cox & Snell *R*^2^/Nagelkerke *R*^2^	0.674/0.992		

A Fisher’s exact test of independence revealed a statistically significant association between occupational exposure risk and HBV vaccination adherence among healthcare workers, *p* < 0.001. As shown in Table 7 (a and b), none (0%) of the workers without exposure were fully vaccinated, compared to 30.3% of those who had experienced occupational exposure.

**Table 7:** Classification table of the logistic regression model predicting Hepatitis B vaccination status among healthcare workers

	Observed		Predicted		
			Vaccination status		Percentage correct
			Incomplete	Fully vaccinated	
Step 1	Vaccination status	Incomplete	392	2	99.5
		Fully vaccinated	0	136	100.0
	Overall percentage				99.6

^a^The cut value is 0.500

A binary logistic regression analysis was conducted to examine the effect of demographic variables on HepB vaccination adherence. Results showed that Gender, Age, Marital Status, Religion, Profession, Department, and Highest Education all showed significant relationships with vaccination adherence (*p* < 0.001). The overall model was significant, *p* < 0.001, explaining 99.6% of the variance in vaccination status.

The model’s predictive power was high, with an overall classification rate of 99.6% (Table 7). Notably, the variable Gender showed no significant effect (*B* = − 205.582, *p* = 0.914), while Age and Department had extreme effects (OR = 6.0 × 10^45^ and OR = 1.3 × 10^52^, respectively), suggesting either data inconsistencies or significant associations.

Model setup was:

- X = DOYOUBEL (digital intervention)
- M1 = KNOWSCRE (Knowledge Score)
- M2 = BARSCORE (Barriers Score)
- Y = VACSTTUS (Vaccination Status)
- Model type = parallel mediation (PROCESS Model 4)
- Sample size = 428

A parallel mediation analysis (Model 4 of PROCESS Macro) was conducted to examine whether the effect of digital intervention belief on HBV vaccine adherence was mediated through knowledge improvement and reduction in perceived barriers (Table 8). The model was significant (McFadden’s *R*^2^ = 0.5667; Nagelkerke *R*^2^ = 0.6336). The total indirect effect of digital intervention on vaccine uptake was significant (effect = − 19.3687, 95% CI [− 22.5504, − 14.7749]).

**Table 8:** Effect of digital intervention on mediators

Outcome variable	Predictor	Coefficient (*B*)	SE	*t*-value	*p*-value	95% CI
Knowledge Score (KNOWSCRE)	DOYOUBEL	− 0.3429	0.0231	− 14.87	< 0.001	[− 0.3882, − 0.2976]
Barrier Score (BARSCORE)	DOYOUBEL	− 0.4048	0.0165	− 24.56	< 0.001	[− 0.4372, − 0.3724]

Higher belief in digital intervention (DOYOUBEL) is linked to lower barriers and unexpectedly lower knowledge scores

Specifically, the indirect effect through perceived barriers was significant (effect = − 19.3377, 95% CI [− 22.5274, − 14.7436]), indicating that digital interventions potentially improve vaccine uptake primarily by reducing perceived barriers to vaccination (Tables 9). The mediation pathway through knowledge was not statistically significant (Effect = − 0.0311, 95% CI [− 0.2947, 0.2570]). There was no significant direct effect of digital intervention on vaccination adherence after controlling for the mediators.

**Table 9:** Effect of mediators on vaccination status

Predictor	Coefficient (*B*)	SE	*Z*-value	*p*-value	95% CI
Knowledge Score (KNOWSCRE)	0.0906	2148.4682	0.0000	1.000	[− 4210.8298, 4211.0111]
Barrier Score (BARSCORE)	47.7670	1810.3579	0.0264	0.979	[− 3500.4694, 3596.0034]

Neither knowledge nor barriers were significant direct predictors of vaccination status individually (*p*-values > 0.05)

**Table 10:** Indirect effects (mediation effects)

Path	Effect	Boot SE	95% Boot CI
Total indirect effect	− 19.3687	2.2423	[− 22.5504, − 14.7749]
Through knowledge (M1)	− 0.0311	0.1378	[− 0.2947, 0.2570]
Through barriers (M2)	− 19.3377	2.2382	[− 22.5274, − 14.7436]

**Table 11:** Direct effect of digital intervention on vaccination

Predictor	Coefficient (*B*)	Standard error (SE)	*Z*-value	*p*-value	95% Confidence interval (CI)
Digital intervention (DOYOUBEL)	9.1793	1481.1154	0.0062	0.995	[− 2893.7535, 2912.1121]

**Table 12:** Summary

Pathway	Mediated	Significant
Digital → barriers → vaccine uptake	Yes	Significant
Digital → knowledge → vaccine uptake	No	Not significant
Direct digital → vaccine uptake	No	Not significant

The total indirect effect is significant because the 95% Boot CI does not include 0; mediation through Barriers is significant, and mediation through Knowledge is not significant (CI includes 0) (Table 10).

The direct effect of digital intervention belief on HepB vaccination adherence was not statistically significant (*B* = 9.179, SE = 1481.115, *p* = 0.995, 95% CI [− 2893.75, 2912.11]) (Table 11). This suggests that digital intervention does not directly influence vaccination adherence after accounting for knowledge and barriers (Table 12).

A moderation analysis using PROCESS Model 1 revealed that gender significantly moderated the relationship between attitude toward HBV vaccination and vaccination adherence among healthcare workers (interaction Chi-square = 38.62, *p* < 0.001) (Tables 13, 14, 15, 16). The overall model was highly significant (Nagelkerke *R*^2^ = 0.8804), indicating strong explanatory power and explains about 88% of the variance in vaccination status. Further conditional effects analysis showed that for females, a more positive attitude was significantly associated with greater vaccine uptake (*B* = − 4.99, SE = 0.95, *p* < 0.001). However, for males, the relationship was not statistically significant (*p* = 0.9302). These findings suggest that attitude toward HBV vaccination has a stronger impact on vaccine adherence among female healthcare workers compared to males.

**Table 13:** Model fit

Metric	Value
−2 Log likelihood (−2LL)	119.93
McFadden's *R*^2^	0.8013
Cox & Snell *R*^2^	0.5986
Nagelkerke *R*^2^	0.8804
p-value (overall model)	< 0.001

**Table 14:** Variables in the model

Predictor	Coefficient (*B*)	SE	*Z*-value	*p*-value	95% CI
Constant	− 13.5474	231.9463	− 0.0584	0.9534	[− 468.15, 441.06]
Attitude Score (ATTSCORE)	− 42.0725	457.0079	− 0.0921	0.9266	[− 937.79, 853.65]
Gender (SEX)	19.1408	391.5018	0.0489	0.9610	[− 748.19, 786.47]
Interaction term (attitude × gender, Int_1)	62.5871	771.3831	0.0811	0.9353	[− 1449.30, 1574.47]

**Table 15:** Moderation test (interaction test)

Test	Chi-square	df	*p*-value
Interaction (Attitude Score * gender)	38.6178	1	< 0.001

The overall interaction test is significant (*p* < 0.001), suggesting that gender significantly moderates the relationship between attitude and vaccine uptake at the model level

**Table 16:** Conditional effects (effect of attitude at different genders)

Gender (sex)	Effect of attitude (*B*)	SE	*Z*-value	*p*-value	95% CI
Male	− 67.5797	771.3825	− 0.0876	0.9302	[− 1579.46, 1444.30]
Female	− 4.9926	0.9468	− 5.2730	< 0.001	[− 6.8484, − 3.1369]

For females, attitude significantly predicts vaccination uptake (*p* < 0.001), whereas for males the effect is not significant

## Discussion

The present study offers a multifaceted exploration into the behavioral, demographic, psychosocial, and digital determinants of Hepatitis B virus (HBV) vaccine adherence among healthcare workers (HCWs) in the study population, with implications for public health strategies in resource-constrained settings. Through a comprehensive analytic framework integrating mediation, moderation, and regression models, this study unravels critical mechanisms underpinning Hepatitis B vaccination behaviors and provides evidence-based recommendations for optimizing vaccine uptake.

Contrary to conventional models such as the Knowledge–Attitude–Practice (KAP) framework, this study found no significant mediation of knowledge on vaccination adherence through attitude [14]. The expectation that increased knowledge would positively shape attitudes and consequently behavior did not hold in this study population. One possible explanation is that, unlike experimental or longitudinal studies where knowledge is deliberately enhanced and tracked, our cross-sectional design captured pre-existing attitudes shaped more by social norms, trust in authorities, and emotional responses than by factual understanding. This aligns with prior research suggesting that knowledge, though important, is often insufficient on its own to shift vaccination behavior [1–3]. While attitude independently predicted vaccine uptake, knowledge had no significant effect on either attitude formation or behavioral outcomes [15]. This finding compels a re-evaluation of health communication strategies that prioritize information dissemination as a cornerstone of behavioral change. In many public health campaigns, especially in the Global South, the assumption remains that knowledge alone suffices to shift attitudes and practices [16–18]. However, although the negligible explanatory power of knowledge in this study indicates that knowledge alone may not sufficiently explain vaccination attitudes, previous research has demonstrated that affective, contextual, and social factors play a stronger role [19–21]. It is possible that in professional environments such as healthcare settings, where baseline awareness of HBV risks is assumed to be relatively high, further increases in knowledge reach a plateau in effectiveness [22–24]. Moreover, vaccine decisions may be filtered through personal beliefs, peer norms, institutional trust, and perceived barriers, factors not sufficiently influenced by knowledge in isolation [25–28].

Despite the weak role of knowledge, attitude toward Hepatitis B vaccination emerged as a potent determinant of vaccine adherence. This reinforces the idea that behavioral interventions must address deeper psychological constructs, including trust, fear, motivation, and perceived benefit or risk [29, 30]. The negative coefficient for attitude indicates a counterintuitive direction of effect, potentially due to reverse coding or the framing of the attitude scale [31, 32]. Nevertheless, its strong association with uptake confirms that attitudinal components, whether positive or negative, are central in shaping vaccination behavior. This is consistent with theoretical models such as the Health Belief Model and the Theory of Planned Behavior, which emphasize perceived susceptibility, severity, and behavioral control as predictors of preventive health actions [33–36]. Future interventions targeting HCWs should therefore consider leveraging attitude-shifting tools, such as testimonial-based messaging, peer-led advocacy, and value-based narratives, rather than focusing exclusively on cognitive or informational strategies [37–39].

One of the most compelling findings is the strong link between occupational exposure risk and Hep B vaccine adherence. None of the HCWs without exposure were vaccinated, whereas a notable proportion of those with documented exposure had completed the vaccination schedule. This suggests that risk perception grounded in lived experience may serve as a more immediate and powerful behavioral driver than abstract knowledge or generalized attitudes [40–42]. This observation is congruent with Protection Motivation Theory, which posits that threat appraisal derived from direct or vicarious experience increases protective behavior [43, 44]. HCWs with no exposure may underestimate their vulnerability, while those who have faced exposure are more likely to adopt preventive measures as a risk mitigation strategy [45]. These findings carry profound implications for public health interventions. Simulation exercises, storytelling from peers who experienced needlestick injuries, or case reviews of post-exposure prophylaxis might be effective in bridging the "perception gap" among unexposed staff, thereby fostering proactive vaccination [46, 47].

Efforts to examine digital access as a moderator of the occupational exposure–vaccination relationship were hindered by a lack of variability in the digital access variable. The vast majority of HCWs reported no access to digital platforms for health-related purposes, pointing to an underutilized domain for behavioral change interventions [48–50]. This finding is critical in an era where digital health strategies are increasingly promoted as scalable, cost-effective tools for behavior modification. The digital divide, whether due to infrastructural, economic, or literacy-related barriers, remains a formidable obstacle to deploying these solutions in low-resource healthcare environments [51–54]. While digital interventions hold theoretical promise, their implementation must be contextualized. Hybrid models that incorporate analog components, e.g., printed infographics, in-person workshops, SMS nudges, may serve as transitional tools in digitally underserved populations [51, 52].

Where digital belief systems were present, their impact on vaccine adherence was found to be mediated by a reduction in perceived barriers rather than through increased knowledge. This unexpected result further emphasizes the limited role of knowledge and redirects focus to the structural or psychosocial impediments HCWs face in accessing vaccination [55, 56]. Digital interventions, when well-designed, may reduce logistical challenges (e.g., appointment scheduling), psychological hesitancies (e.g., fear of side effects), or procedural ambiguities (e.g., where to get vaccinated) [56, 57]. However, they must be tailored to target these barriers directly rather than assuming that mere exposure to digital content will automatically improve health literacy or behavior [56]. Interestingly, belief in digital intervention was negatively associated with knowledge, raising questions about content quality, credibility, and user experience. In settings where misinformation proliferates, digital platforms may inadvertently contribute to confusion or skepticism. Ensuring evidence-based content, verified sources, and culturally resonant messaging is essential if digital strategies are to succeed in this context [58–60].

The logistic regression analysis exploring the effects of demographics on vaccination adherence produced a statistically significant model with exceedingly high explanatory power. However, individual coefficients, especially for age and department, were implausibly large, hinting at potential model instability, multicollinearity, or overfitting. Despite this, the statistical significance of key demographic variables reinforces the idea that vaccine behavior is not merely a product of individual psychology but is embedded within broader social, professional, and institutional ecosystems [61]. For example, departments with more rigorous infection control policies may enforce vaccination adherence, while educational background might reflect differential access to immunization information or professional expectations [61]. The non-significant effect of gender in this model, contrasted with its moderating role in attitude–behavior relationships, also illustrates the context-specific nature of demographic effects. Gender may not directly predict vaccine uptake but can shape the processes by which psychosocial constructs such as attitude exert influence [62].

The moderation analysis showed that attitude significantly predicted vaccine uptake among females but not among males. This finding highlights a nuanced gendered pathway through which beliefs and behaviors interact [62]. It suggests that female HCWs may be more attuned or responsive to attitudinal shifts in shaping health behavior, whereas male HCWs may be influenced by other factors such as organizational mandates, peer norms, or personal risk assessment. These differences may reflect sociocultural norms, professional hierarchies, or communication preferences. In many settings, female HCWs often engage more actively in peer support networks and may be more exposed to affective messaging, thereby translating attitude into behavior more readily. For policy and programming, this implies the need for gender-sensitive intervention designs. Messaging that appeals to professional identity, authority, or personal responsibility may be more effective for male HCWs, whereas community-building, empathy-based appeals may resonate more with female staff. One-size-fits-all campaigns are unlikely to yield uniform results in diverse healthcare workforces [63, 64].

## Limitations of the study

The present study’s use of multiple PROCESS models allows for a granular dissection of mediation and moderation pathways, offering a layered understanding of the dynamics at play. However, several limitations must be acknowledged. First, the disproportionate absence of digital access among participants limits generalizability regarding the digital ecosystem’s influence on vaccine uptake. Second, extreme values in regression coefficients suggest the presence of collinearity or model misspecification, necessitating replication and robustness testing. Third, the measurement of constructs such as attitude, knowledge, and perceived barriers, though statistically modeled, must be carefully validated to ensure construct reliability and conceptual clarity. The inverse directionality of attitude effects, for example, warrants further qualitative exploration. Furthermore, the inclusion of “belief in digital intervention” as a predictor represents a conceptual limitation. While digital tools are emerging within Nigeria’s broader e-health landscape, there is currently no adequately documented evidence of HBV-specific digital interventions. As such, the construct should be interpreted as exploratory and forward-looking rather than reflective of existing infrastructure, which may limit the validity of conclusions drawn from this pathway. An additional limitation relates to the study’s eligibility criteria and sampling of healthcare worker cadres. While the study included a broad range of professionals, an omission occurred in the questionnaire’s profession section, where physiotherapists were not explicitly listed. Although the “Others” category captured this group, the lack of explicit representation reduces the generalizability of the findings, as not all healthcare worker cadres were distinctly accounted for. Lastly, this study’s cross-sectional nature precludes causal inferences. Longitudinal studies or randomized trials will be critical in confirming pathways and testing the effectiveness of targeted interventions.

## Conclusion

This study provides compelling and robust evidence that Hepatitis B virus (HBV) vaccine uptake among healthcare workers (HCWs) is influenced more by affective, experiential, and contextual factors than by cognitive knowledge alone. While knowledge is important, it is insufficient on its own to drive vaccine adherence. Attitudes, perceived risks and barriers, previous occupational exposures, and gender-related dynamics are identified as critical determinants shaping vaccine behavior. Despite the potential of digital health interventions to enhance uptake, their promise remains largely unrealized due to infrastructural limitations, digital illiteracy, and unequal access. Therefore, health systems must shift from traditional, knowledge-centered approaches toward a more holistic and evidence-based strategy. This strategy should include behaviorally informed program design, ensure digital inclusion, promote gender-sensitive messaging, and foster institutional accountability and engagement. Only through the implementation of such multifaceted, contextually grounded interventions can vaccine adherence among HCWs, who form the frontline defense against infectious disease threats, be sustainably improved.

## Supplementary Information

Supplementary material 1.

## Data Availability

The datasets generated and analyzed during the current study are not publicly available due to privacy considerations of the participants, but are available from the corresponding author upon reasonable request.
