# Supplementary material for: Psychosocial and digital predictors of Hepatitis B vaccination uptake in healthcare workers: insights from a Nigerian tertiary hospital
Source: Trop Med Health. 2025 Dec 31;53:198. doi: 10.1186/s41182-025-00893-4 (PMC12754984; doi:10.1186/s41182-025-00893-4)
Supplement: Supplementary file 1 — Supplementary material 1. [file 41182_2025_893_MOESM1_ESM.docx]

**Questionnaire**
Dear respondent,
Thank you for taking the time to complete this questionnaire. Your responses will contribute to the research on health professionals at FMC Keffi. Please tick (✓) the appropriate boxes or provide details where required. All information will be kept strictly confidential.

**Section 1: Personal Information**

**1. Sex**
☐ Male
☐ Female

**2. Age (years)**
☐ <20

☐ 20–30
☐ 31–40
☐ 41–50
☐ 51–60
☐ >60

**3. Marital Status**
☐ Single
☐ Married
☐ Divorced

**4. Religion**
☐ Christianity
☐ Islam
☐ Traditional Religion
☐ Others (please specify): _____________

**Section 2: Professional Information**

**5. Profession**
☐ Radiologist

☐ Nurse
☐ Midwife
☐ Lab Technician
☐ General Practitioner
☐ Dental Doctor
☐ Pharmacist
☐ Specialist/Consultant

☐ Other

**6. Working Department**
☐ Inpatient Department
☐ Outpatient Department
☐ Emergency Department
☐ Delivery Unit
☐ Laboratory
☐ OR (Operating Room) Department
☐ Dental Department

**7. Work Experience**
☐ <5 years
☐ ≥5 years

**8. Have you received training on infection prevention?**
☐ Yes
☐ No

**9. Highest Educational Qualification**
☐ OND (Ordinary National Diploma)
☐ HND/BSc/MBBS
☐ MSc
☐ Fellowship/PhD/MD

**Section 3: Attitudes and Beliefs**

Please indicate your level of agreement with the following statements:

| **Statement** | **Strongly Agree** | **Agree** | **Neutral** | **Disagree** | **Strongly Disagree** |
| --- | --- | --- | --- | --- | --- |
| HBV is a serious public health problem | ☐ | ☐ | ☐ | ☐ | ☐ |
| All patients should be tested for HBV before they receive healthcare | ☐ | ☐ | ☐ | ☐ | ☐ |
| Being a health professional puts you at greatest risk of HBV infection | ☐ | ☐ | ☐ | ☐ | ☐ |
| Following infection control guidelines will protect me from being infected with HBV and HCV | ☐ | ☐ | ☐ | ☐ | ☐ |
| I deliver the same standard of care to patients with HBV as I do for other patients | ☐ | ☐ | ☐ | ☐ | ☐ |
| It is appropriate not to spend much time when caring for HBV-infected patients | ☐ | ☐ | ☐ | ☐ | ☐ |
| A healthcare worker can infect patients with HBV | ☐ | ☐ | ☐ | ☐ | ☐ |
| Health professionals who are HBV-positive should not give healthcare services to patients | ☐ | ☐ | ☐ | ☐ | ☐ |
| I do not trust the HBV vaccine | ☐ | ☐ | ☐ | ☐ | ☐ |
| HBV vaccine should be compulsory | ☐ | ☐ | ☐ | ☐ | ☐ |
| HBV vaccine is safe but expensive | ☐ | ☐ | ☐ | ☐ | ☐ |
| After exposure to contagious fluid/material, the vaccine reduces the likelihood of HBV infection | ☐ | ☐ | ☐ | ☐ | ☐ |

**Section 4: Occupational Exposure**

**1. Have you experienced occupational exposure to HBV risk?**
☐ Yes ☐ No

**2. Have you experienced a sharp injury at work?**
☐ Yes ☐ No

**3. Have you had unprotected mucocutaneous fluid contact on intact skin?**
☐ Yes ☐ No

**4. Have you had body fluid contact through body openings (e.g., eyes, mouth, etc.)?**
☐ Yes ☐ No

**Section 5: Post-Exposure Measures**

**1. What measures did you take after exposure? (You may tick more than one option)**
☐ Testing the patient right away
☐ Washing with soap, water, or antiseptic
☐ Immediate report to authority
☐ Allowing the injury area to bleed
☐ Waited and tested myself later

**Section 6: HBV Screening and Vaccination**

**1. Have you ever been screened for hepatitis B?**
☐ Screened
☐ Not screened

**2. Have you been vaccinated for hepatitis B?**
☐ Vaccinated
☐ Not vaccinated

**3. If vaccinated, how many doses have you received?**
☐ Once only (incomplete vaccination)
☐ Two doses (incomplete vaccination)
☐ Three doses (fully vaccinated)

**4. What is your vaccination status?**
☐ Fully vaccinated
☐ Incomplete vaccination

**5. Have you been tested for vaccine effectiveness after completing the full dose?**
☐ Tested for the vaccine effect
☐ Not tested

**6. If tested, what was the result?**
☐ Protected (anti-HB titer > 10MIU/ml)
☐ Not protected

**Section 7: Reasons for Incomplete or No Vaccination**

**1. If your vaccination is incomplete, what is the reason? (You may tick more than one option)**
☐ Being busy
☐ I feel I am protected
☐ Forgot it completely
☐ Waiting for the next dose

**2. If you did not take the vaccine, what was the reason? (You may tick more than one option)**
☐ The vaccine was not available through government channels
☐ The vaccine is very expensive for private access
☐ I did not give it much emphasis
☐ I was concerned about possible side effects
☐ The duration of total doses is too long
☐ Others (please specify): _____________

| **Ser. No.** | **Questions** | **Responses** | **Skips** |
| --- | --- | --- | --- |
| **1** | Have you taken special training about HBV? | ☐ Yes (1) |  |
|  |  | ☐ No (2) |  |
| **2** | Have you got any medical advice about HBV? | ☐ Yes (1) |  |
|  |  | ☐ No (2) |  |
| **3** | Have you seen patients who have HBV? | ☐ Yes (1) |  |
|  |  | ☐ No (2) |  |
| **4** | Do you want to pay for hepatitis B vaccination? | ☐ Yes (1) → Skip to **6** |  |
|  |  | ☐ No (2) |  |
| **5** | Why do you not want to pay? | ☐ The vaccine is not available (1) |  |
|  |  | ☐ I am not at risk of acquiring the virus (2) |  |
|  |  | ☐ I am not aware that the vaccine is available (3) |  |
|  |  | ☐ My friends are not willing to pay (4) |  |
|  |  | ☐ I do not have time for vaccination (5) |  |
|  |  | ☐ The vaccination process is time-consuming (6) |  |
|  |  | ☐ The vaccine is not effective (7) |  |
|  |  | ☐ Others (specify) (96) |  |
| **6** | If you want, will you pay **15,000 NGN** per dose? | ☐ Yes (1) |  |
|  |  | ☐ No (2) |  |
| **7** | What if the price is **30,000 NGN** per dose? | ☐ Yes (1) |  |
|  |  | ☐ No (2) |  |
| **8** | What if the price is **20,000 NGN** per dose? | ☐ Yes (1) |  |
|  |  | ☐ No (2) |  |
| **9** | If you really want to buy, what is the **final minimum amount** you are willing to pay per dose? | ________________________ **NGN** | Stop the interview here. |
| **10** | What if the price is **50,000 NGN** per dose? | ☐ Yes (1) |  |
|  |  | ☐ No (2) |  |
| **11** | What if the price is **75,000 NGN** per dose? | ☐ Yes (1) |  |
|  |  | ☐ No (2) |  |
| **12** | What if the price is **100,000 NGN** per dose? | ☐ Yes (1) |  |
|  |  | ☐ No (2) |  |
| **13** | Due to inflation or other uncertainties, what is the **maximum amount** you are willing to pay per dose? | ________________________ **NGN** |  |

**Do you think digital tools (e.g., mobile apps, SMS reminders) can increase vaccine uptake?**
☐ Strongly Agree
☐ Agree
☐ Neutral
☐ Disagree
☐ Strongly Disagree

**Have you previously used digital platforms to manage your health-related tasks (e.g., reminders, records)?**
☐ Yes
☐ No

**Section 9: Barriers to Vaccine Uptake**

**What challenges do you think prevent healthcare workers from getting vaccinated? (Select all that apply)**
☐ Lack of awareness
☐ Cost of the vaccine
☐ Lack of time
☐ Fear of side effects
☐ No reminders or follow-ups
☐ Other (please specify): _______________

**Do you believe digital interventions can address these barriers?**
☐ Yes
☐ No
☐ Not Sure

**Section 10: Digital Interventions**

**Which type of digital intervention do you think would be most effective for increasing vaccine uptake? (Select all that apply)**
☐ SMS reminders for vaccination schedules
☐ Mobile apps for vaccine education and tracking
☐ Telemedicine consultations for vaccine counseling
☐ Online training programs on Hepatitis B prevention and vaccination
☐ Social media campaigns targeted at healthcare workers
☐ Other (please specify): _______________

**How often would you prefer to receive digital reminders for vaccination?**
☐ Weekly
☐ Bi-weekly
☐ Monthly

**What type of information would you like digital tools to provide? (Select all that apply)**
☐ Benefits of the Hepatitis B vaccine
☐ Vaccination schedule reminders
☐ Locations offering free or subsidized vaccines
☐ Testimonials from other healthcare workers
☐ Side effects and safety information

**What devices do you typically use to access digital information? (Select all that apply)**
☐ Smartphone
☐ Tablet
☐ Computer
☐ Other (please specify): _______________

**Section 11: Feedback and Suggestions**

**Would you recommend incorporating digital interventions into FMC, Keffi's vaccination campaigns?**
☐ Yes
☐ No

**What specific features would you like to see in a digital intervention for Hepatitis B vaccination?**

**Any other suggestions or comments?**

**THANK YOU VERY MUCH!!!**
